# A Case of Live Birth after Uterine Reconstruction for Recurrent Cornual Ectopic Pregnancy following IVF Treatment

**DOI:** 10.1155/2013/625261

**Published:** 2013-02-12

**Authors:** Deivanayagam Maruthini, Vinay Sharma

**Affiliations:** The Leeds Centre for Reproductive Medicine, Seacroft Hospital, Leeds LS14 6UH, UK

## Abstract

We present a case of recurrent ruptured right cornual ectopic pregnancies conceived after IVF. Following the second episode, a sonohysterography was undertaken to identify possible areas of scar weakness that may rupture with uterine distension in a future pregnancy. The scan revealed asymmetrical muscle thickness in the cornual regions, the right (6 mm) being thinner than the left (1.6 cm). Subsequently, an elective laparotomy was undertaken, and the cornua were reconstructed and thickened in several layers by bringing the laterally retracted myometrial fibres onto the reconstruction site. A sono-hysterography after surgery showed satisfactory (3-4 cm) myometrial thickness all around. A further cycle of IVF resulted in a singleton pregnancy. Pelvic scans confirmed normal intrauterine pregnancy without any myometrial thinning. She was delivered by an uneventful elective caesarean section at term. We propose that, in those who intend to have further pregnancies after a cornual ectopic pregnancy, a sono-hysterography is possibly the best investigative tool to assess myometrial integrity. This case demonstrates that in women with areas of muscle weakness it is possible to successfully perform an interval elective reconstructive surgery on the uterus that can result in an uneventful pregnancy and birth.

## 1. Introduction

Cornual (interstitial) ectopic pregnancy is a life-threatening complication especially if it ruptures before diagnosis and significant intraperitoneal bleeding occurs. Mayer et al. [1] has highlighted the difficulty in differentiating an intrauterine from an extrauterine pregnancy, particularly with reference to the angular location of the gestational sac within the cornual region. A medline search for literature on recurrent cornual ectopic resulted in only one publication that based on their experience in a single institution, estimated this risk to be 0.3% whilst reporting their 4 recurrent cornual ectopics in 53 index cases [2]. This was a report on 4 cases that described laparoscopic management of recurrent ectopic gestations [2]. True incidence of recurrent cornual ectopic is not known for most women after a rupture is advised against a further pregnancy because of the risk of uterine scar dehiscence in pregnancy and its complications. Many women will also have tubal disease and may thus be unable to conceive spontaneously, and some will resort to surrogacy in those circumstances. The aim of this report is to highlight the role of further assessment of uterine integrity after recurrent cornual ectopic pregnancy but prior to any future conception. Our suggestion for further evaluation of uterine integrity is supported by a case report of recurrent ruptured cornual ectopic pregnancy in which a sonohysterography followed by an elective novel uterine reconstruction surgery resulted in a live birth.

## 2. Case Presentation

A 33-year-old woman and her 30-year-old husband presented with history of primary infertility over a period of 4 years. Although she did not report a history of pelvic inflammatory disease or sexually transmitted infection in the past, she had ultrasound confirmed bilateral hydrosalpinges, and both of her fallopian tubes had been removed laparoscopically. Thereafter, she required fertility assistance by *in vitro* fertilization (IVF). 

After confirmation of a pregnancy in the 1st attempt of IVF in February 2005 she was admitted to the local hospital with abdominal pain during the first trimester. Following an ultrasound suspicion of an interstitial pregnancy, she underwent a laparoscopy, which confirmed a right cornual ectopic pregnancy. The operation was then graduated to a laparotomy, and the cornual ectopic was removed by a longitudinal incision on the right cornu. After evacuating the products, the bleeding was minimised by diathermy coagulation. Postoperatively, she received supplementary treatment with intramuscular methotrexate to resolve any remnant trophoblastic tissue. Trophoblastic tissue, however, remained viable for up to 3 months after the first dose of methotrexate, and serum hCG was detectable albeit at low concentrations (27–45 iu/L). She opted for a conservative management as opposed to a repeat dose of methotrexate, and it was finally 8 months before all evidence of trophoblastic activity disappeared completely. 

She conceived again after the 2nd attempt of IVF performed in February 2006. This time she presented with an acute abdomen at 6-week gestation at her local hospital and underwent an emergency laparoscopy. She was then found to have 1200 mL of haemoperitoneum. Active bleeding at the right cornu was seen, but trophoblastic tissue attached to the uterine cornu was not seen. Subsequently tissue found amongst the blood clots was histologically confirmed to be the “product of conception.” Due to this very early rupture, the exact location of this pregnancy and muscular integrity of the uterine cornua could not be assessed by scan prior to the rupture. The surgeon used a laparoscopic approach to achieve haemostasis. A blunt diathermy probe was inserted into the cornu, and the cornual bleeding site was coagulated from outside to inside. It was hoped that the diathermy coagulation would also lead to occlusion of the interstitium.

Upon recovery the patient returned to the tertiary centre for assisted conception. She then had a detailed discussion with regards to the possible causes and consequences of recurrent cornual ectopic pregnancies. The patient was advised of the high risk of recurrent uterine rupture in another pregnancy with potentially lifethreatening consequences. Since she was strongly motivated to give birth to a child, a couple of options were discussed with her as below.

- Hysteroscopic occlusion of the intramural part of the fallopian tubes was considered. At that time, this method was being practiced only for sterilisation by a single surgeon at our hospital. It had not gained popularity due to its high failure rate. There was no evidence for its value in preventing an interstitial pregnancy, and there could have been the risk of an adverse impact on pregnancy and uterine perforation with the ring coils. Recurrent uterine dehiscence due to primary weakness in the myometrium at this cornual site remained a concern, and hence this was not considered a sufficiently safe option.
- Assessment of uterine integrity as a first step followed by uterine repair if deemed necessary without any intervening myometrium in between the serosa and the uterine cavity. She was made aware that the proposed operation was novel to the author's knowledge and had not been done before. Therefore no information could be given regarding the likelihood of its success and the risk of uterine rupture in a future pregnancy. She consented to this strategy after receiving ample opportunity to reconsider and reflect.

A preliminary ultrasound scan of her pelvis showed a normally appearing uterine fundus without any evidence of asymmetry of either cornu. A further assessment of myometrial integrity at the site of uterine rupture was deemed necessary. After appraisal of the available investigative techniques a saline sonohysterography as a real time assessment of the myometrial thickness was chosen as the method of choice. A saline sonohysterography was performed, whereby the uterine cavity was sealed at the cervix with a sonography balloon catheter (H/S catheter set with ingrated stylet, Cooper Surgical, CT 61-3005 5F), and the uterus was subjected to gradual distension with warmed normal saline. This was carefully and slowly injected to avoid myometrial spasm until the patient experienced discomfort. At this point the scan frame was frozen, and the distension reduced. This process was slowly repeated several times in the same episode to enable multiple measurement opportunities and to minimise intraobserver variation. The minimum myometrial thickness was measured in the transverse plane at the uterine fundus on both sides, and this revealed an asymmetry of muscle thickness in the cornual regions. The myometrial thickness in the left cornual region was 5-6 mm whilst, it was less than half of this thickness at 1.6 cm on the contralateral side. Additionally with saline distension, during real time scanning, a track of endometrial cavity on the right side with suspected fluid was seen leaking through the track into the peritoneal cavity suggesting thereby the possibility of an endometrial-peritoneal fistula.

A further detailed outpatient discussion with the patient and partner occurred in April 2006 and increased risk of another acute uterine rupture during pregnancy at this cornual site or a slow rupture leading to abdominal pregnancy was discussed. The couple were advised to consider the alternatives of surrogacy and/or adoption. However, the patient remained strongly motivated to try an elective uterine reconstruction. Therefore an elective laparotomy was performed.

**Figure 1:**
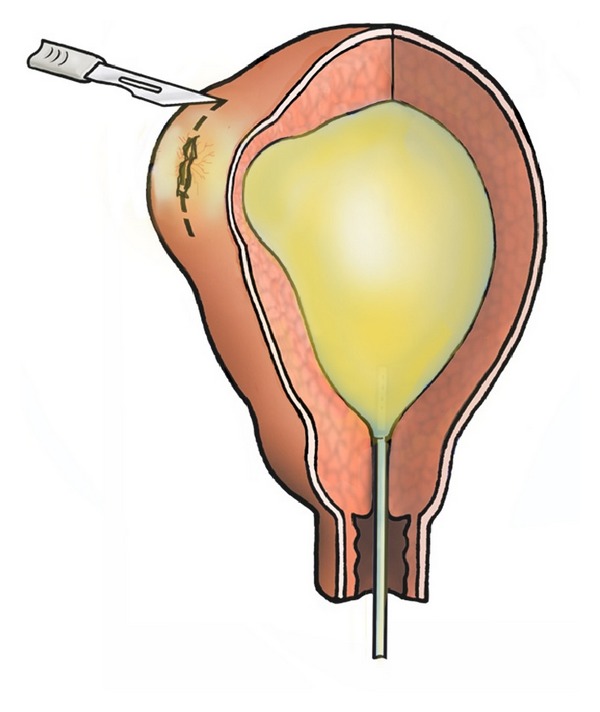
A size 10 Foley's catheter was inserted into the uterine cavity, distended with 10 mL of methylene blue, and left in situ. At laparotomy, neovascularisation that radiated like spider nevi was observed in the right cornu where only the serosal surface of the uterus separated the intrauterine balloon without any intervening myometrium. It was evident that this site would have been the site of previous cornual rupture forming an endometrial-peritoneal fistula.

**Figure 2:**
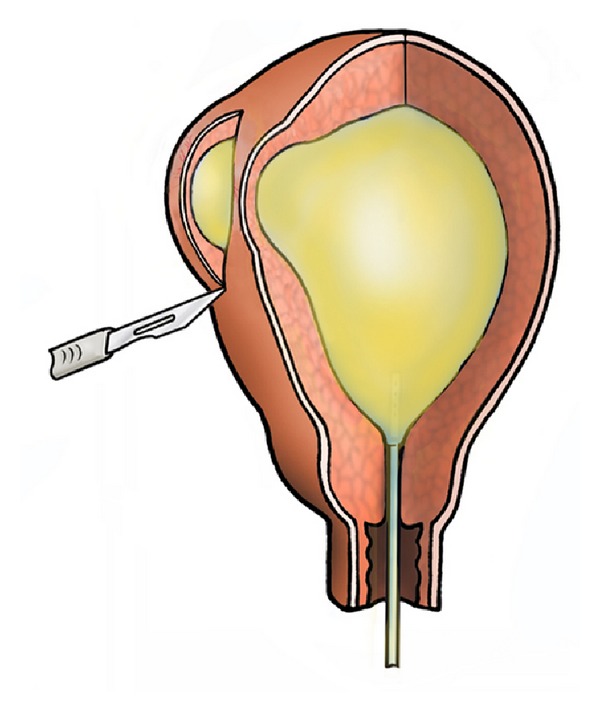
An incision along the right cornu immediately reached the endometrial cavity at a depth of 2.5 mm revealing extremely thin uterine wall.

At surgery in July 2006, a hysteroscopy was performed in the first instance, and this showed a normal uterine cavity. No abnormalities were noted in the cornual regions. A unique surgical method to identify areas of myometrial weakness was then devised. A 10 mL balloon Foley's catheter, size 10, was inserted into the uterine cavity, the balloon was gently distended with warm methylene blue, and the catheter was left in situ. A laparotomy was performed immediately, and uterus carefully inspected. The uterus externally appeared normal. Although both uterine cornua were found to be thin, in the right cornual region, there were two sites at which only peritoneum separated the intrauterine balloon and the serosal surface of the uterus, and methylene blue was seen through the peritoneum without any intervening myometrium between the layers (Figure 1). Adjacent to this site was a vesicular blue lesion that resembled an endometriotic nodule with neovascularisation that radiated like spider nevi. At this site also the uterine muscle layer was extremely thin, and upon incision around the nodule the intrauterine catheter was reached at the depth of 2.5 mm (Figure 2). It was evident that these sites of weakness would have been the site of previous cornual ectopic and uterine rupture and the endometrial-peritoneal fistula formation. 

**Figure 3:**
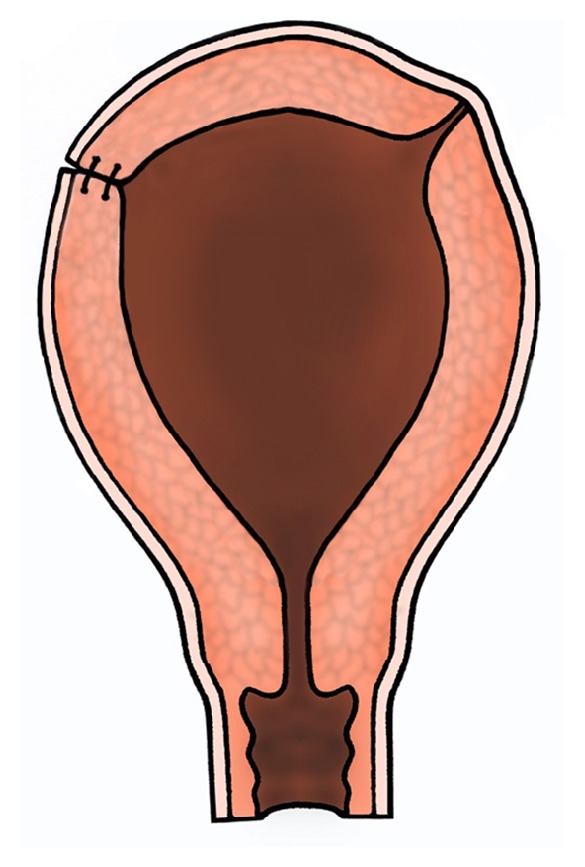
The cornu was gradually reconstructed by 2-3 layers of continuous suturing with vicryl (polyglactin 910) sutures. The cornual musculature was artificially thickened by bringing the laterally retracted deep myometrial fibres from around the cornua onto and above the incision site.

**Figure 4:**
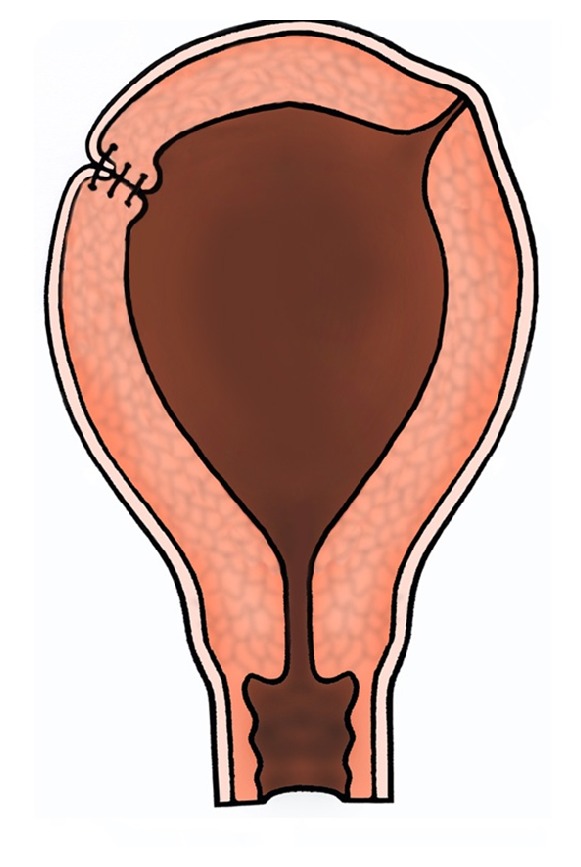
Inverting superficial polypropylene sutures were applied to the serosal layers overlaying the uterine muscle to prevent fibrosis or adhesion formation on the peritoneal surface.

The uterine cornu was incised, and the endometrial cavity was immediately entered. The blue nodule was excised, and on inspection of the endometrial cavity it was noted that subsequent to the previous ruptures the deeper muscle layers had retracted into the endometrial cavity. These were brought into the incision site with U-shaped continuous sutures. The cornu was gradually reconstructed in several layers (at least 3), and cornual musculature was artificially thickened by bringing the laterally retracted myometrial superficial fibres from the fundus also onto and above the incision site (Figure 3). Once again U-shaped continuous sutures in several layers with intramural vicryl (polyglactin 910) suture material were used. Inverting superficial prolene (polypropylene) sutures were applied to the uterine muscle on the serosal surface for haemostasis and to prevent fibrosis or adhesion formation on the peritoneal surface (Figure 4). Similar strengthening of the cornu was performed on the left side but without a uterine incision or exposure of endometrium. For this, U-shaped continuous sutures in several layers were placed in the seromuscular part of the thinned-out left cornual region before placing a final inverted superficial prolene suture on the peritoneal surface. She recovered well from the surgery.

At approximately 3 months after the surgery the outpatient saline sonohysterography was repeated in the same manner as described above. On this occasion, myometrial thickness in the transverse plane was assessed; a 3-4 cm symmetrical all round myometrial thickness was noted, particularly at both cornual ends. 

After appropriate discussion and consenting process a fresh cycle of IVF was performed in July 2007. A single embryo was transferred electively on day 3 of embryonic development. Supernumerary embryos were cryopreserved on this day. The pregnancy test was positive, and a scan confirmed a viable intrauterine pregnancy at 7-week gestation. The pregnancy sac was very much at the centre of the uterine cavity without any evidence of asymmetric thinning of the myometrium at the cornua or at any site. The muscle thickness was 4 cm. A further scan at 10 weeks was similarly reassuring with regards to the uterine muscle thickness all around the pregnancy and especially in the cornual areas. The patient was thereafter discharged to the care of her local obstetrician who monitored her closely throughout the pregnancy. She remained well and was delivered by an elective caesarean section at term in 2008 at which time the cornual regions were inspected and found to be normal and intact as in a normal uterus. 

The patient subsequently attended our centre again in 2009 for a frozen embryo transfer cycle to use the spare embryos from the previous attempt. As per patient request, all frozen embryos from her previous IVF attempt were thawed and cultured for 48 hours. A single blastocyst was transferred, but on this occasion a pregnancy was not achieved. This patient has not returned for further treatment. 

## 3. Discussion

Cornual pregnancy is the most dangerous form of ectopic gestation that upon spontaneous rupture can result in severe hypovolemia and circulatory arrest [3]. Even though this is a life-threatening condition, there is no consensus on effective management, subsequent advice, or followup. Sporadically, clinicians have reported the use of hysteroscopic tubal occlusion to reduce the risk of recurrence but not in sufficient numbers for it to be a reliable evidence, and in any case this method aims to prevent all future pregnancies rather than an individual risk assessment [4]. 

Saline sonohysterography was chosen in this case, as it is a dynamic test with the potential to assess the shape of the uterine cavity, myometrial thickness with its functional integrity at the same time. Hysterosalpingography allows assessment of the uterine cavity but is inadequate to measure the thickness of the myometrium in the cornual region, as serosal surface of the uterus is not visible. Hysteroscopy is a subjective assessment and prone to errors in interpretation of uniformity of the cavity. In particular it will not allow any assessment of muscular thickness, areas of weakness, and fistulas to the peritoneal cavity or areas of leakage of fluid, which are important in the assessment of myometrial integrity. Magnetic resonance imaging is an alternative as long as the myometrial interface with the endometrial cavity on one side and peritoneal surface on the other can be accurately defined. However, apart from the expense, MRI will not identify the presence of utero-peritoneal fistula. A 3-D ultrasound scan is less expensive than an MRI scan and is not associated with the same amount of discomfort as with a saline sonography. Thus, a 3-D scan *per se* may form an useful tool to assess the myometrial thickness at various locations of the uterus. However, the potential space within the cornua can only be distended with the help of saline in order to accurately measure the thickness of the myometrium all around the cornual region. A combination of the 3-D technology and the saline sonography may provide a better assessment of the cornual region than the 2-D saline sonography, nevertheless demands expertise for reliable interpretation of the images. Therefore saline sonography is possibly the investigation of choice as close to a physiological assessment as is possible for myometrial integrity upon uterine distension and enlargement with a pregnancy.

In treatment, several strategies are variably employed, and these are generally based upon clinical factors, such as gestational age at the time of diagnosis, viability of the fetus, location of the gestational sac within the uterine cornua, and the myometrial thickness between the conceptus and the serosa. The choice of surgical treatment also depends upon the attending gynaecologist's experience. Patient's wishes and compliance for followup also play a key role in management. Options in use include single-dose methotrexate, high-dose methotrexate, laparoscopic injection of methotrexate into the ectopic gestational sac, laparoscopic excision and repair, open removal of ectopic pregnancy, and even a hysterectomy [5]. 

In the surgical management of cornual gestation, both, laparoscopic and open approaches have been described [6–8]. Laparoscopic management has been popularised in certain centres, but whether or not this is optimally undertaken depends on available surgical expertise in emergency and haemodynamic stability of the patient at presentation [6, 9, 10]. 

For peroperative haemostasis, some surgeons have proposed the use of diathermy, prior to making an incision in the cornua and also following removal of conceptus. However, when performing a myomectomy, the risk of uterine rupture has been reported to be higher if diathermy is the primary tool for haemostasis [11]. In this patient, whether the use of extensive coagulation during the surgical management of two previous cornual ectopic pregnancies might have contributed to the development of subsequent scar weakness, and fistula formation is a subject to be carefully evaluated. Further research is required to prove the causal relationship between diathermy coagulation and irreparable myometrial weakness. Some laparoscopic surgeons have reported the successful use of intramyometrial vasopressin prior to incision in the cornua. 

Comparisons between various types of treatment, risk of recurrent uterine rupture, methods of assessment, and so forth are needed in the development of standardised management. At present it is neither possible to compare the outcomes of various treatment modalities in cornual ectopic pregnancy, nor is it possible to make recommendations regarding most effective treatments. The definition of “successful treatment” and the measure of success itself have varied in different reports, which makes valid comparisons difficult. Furthermore, majority of the reports on method of treatment focus on the immediate surgical outcome, and very few have addressed the long-term consequences such as recurrence of cornual implantation in future pregnancies, risk of recurrent uterine rupture, and the incidence of live birth [12, 13]. This may be so because the incidence of cornual ectopic is extremely low, risk of recurrence is lower still, and subsequent voluntary or involuntary infertility based on medical advice may affect the long-term assessments. 

Furthermore, cornual implantation of a future pregnancy and the myometrial weakness resulting from a previous cornual ectopic pregnancy may not be two mutually exclusive risk factors predisposing the woman for uterine rupture during recurrent cornual ectopic pregnancy. In this patient, thinning of the cornual myometrium in comparison to the rest of the fundal myometrial thickness was taken as an indication of myometrial weakness. Additionally, the presence of a demonstrable utero-peritoneal fistula confirmed her risk of rupture in a future pregnancy. It would be ideal if a “cut off” value for the myometrial thickness could be defined as “safe” for another pregnancy, or below which a recommendation for uterine reconstruction could be made. However, this needs a carefully designed, adequately sized, prospective followup study of index cornual ectopic pregnancies, and validation of myometrial integrity after different modalities of treatment. 

As far as we are aware there has been one recent case report describing the surgical treatment of recurrent cornual ectopic pregnancy [2]. Laparoscopic cornuostomy or wedge resection was recommended by the authors, the former if the ectopic was less than 3 cm in diameter and the latter if the ectopic was more than 3 cm in diameter. The authors described laparoscopic application of a single layer of mattress sutures to the myometrial defect using braided polygalactin 910 or monofilament poliglecaprone 25 sutures. In the abovementioned case series, diathermy was used to a minimum, and haemostatic sutures were preferred for the incidence of uterine rupture after myomectomy has been reported more commonly after the use of diathermy. In this case series, 2 out of 4 cases had successful live births after 36 weeks of gestation following spontaneous or IVF conception.

We place a greater emphasis on multilayer as opposed to single-layer muscle suturing in the reconstruction of the myometrial layer. In this case, cornual reconstruction has to be, such that the risk of uterine rupture in a future pregnancy is minimized, and for this reason we favour the open approach. Multilayered suturing of myometrium using braided, absorbable, and coated polygalactin (vicryl) provides mechanical strength to the area. Additionally, inverting monofilament, nonabsorbable polypropylene (prolene) sutures to the serosa, minimises the risk of adhesions by being an inert material, by providing excellent haemostasis and by reducing the length of the scar as it draws the elastic but strong muscle fibres over the site of incision to replace or coexist with fibrosis. Fibrous tissue is generally less strong than the muscle. This successful outcome demonstrates the ability of the myometrium to remodel and undergo physiological changes during a pregnancy after cornual reconstruction after an ectopic, much in the same way as after a myomectomy or caesarean section, and makes it possible for women to conceive and give birth even after a ruptured ectopic pregnancy. However, the risk of rupture, particularly during labour, at least theoretically remains high until more evidence emerges, and until then careful monitoring during pregnancy and an elective caesarean section for delivery are recommended. 

## 4. Conclusion

Optimal management of an index cornual ectopic gestation is important to minimise the risk of recurrence and uterine rupture in future pregnancies. Such optimal treatment modality is yet to be defined, and currently robust data are lacking with regards to recurrent uterine rupture or live birth in the event of a pregnancy after a ruptured cornual ectopic, spontaneously or after IVF. There is also no guidance in the assessment of myometrial integrity at the site of rupture and no objective way of advising patient of her individual risks and/or corrective measure such as interval elective reconstructive surgery. We propose that appropriate followup and further evaluation of uterine integrity should be routinely undertaken following treatment of cornual ectopic pregnancy in those women who intend to have further pregnancies. This is so that they can receive objective advice on their specific risks, and a reconstructive surgery can be employed when indicated. In this group, saline sonohysterography is possibly the best investigative tool amongst the available modalities for its dynamic nature allowing identification of possible areas of scar weakness that may rupture with uterine distension/enlargement in pregnancy. Further in women with areas of muscle weakness or reduced integrity, this case demonstrates that it is possible to successfully perform an interval elective reconstructive surgery on the uterus that can result in an uneventful pregnancy and birth.
